# *N*′-(5-Hydr­oxy-2-nitro­benzyl­idene)-2-methoxy­benzohydrazide

**DOI:** 10.1107/S1600536809043840

**Published:** 2009-10-28

**Authors:** De-Suo Yang

**Affiliations:** a Department of Chemistry and Chemical Engineering, Baoji University of Arts and Sciences, Baoji 721007, People’s Republic of China

## Abstract

The asymmetric unit of the title compound, C_15_H_13_N_3_O_5_, contains two independent mol­ecules. Each mol­ecule displays an *E* configuration with respect to its C=N double bond. The dihedral angles between the two benzene rings are 11.1 (2) and 10.9 (2)° in the two mol­ecules. In the crystal structure, mol­ecules are linked through inter­molecular O—H⋯O hydrogen bonds, forming chains running along the *a* axis.

## Related literature

For the biological and structural chemistry of hydrazone compounds, see: Avaji *et al.* (2009 ▶); Charkoudian *et al.* (2007 ▶); Cukurovali *et al.* (2006 ▶). For related structures, see: Yang (2008*a* ▶,*b* ▶,*c* ▶,*d* ▶,*e*, 2007*a* ▶,*b* ▶,*c* ▶); Yang & Guo (2006 ▶). For reference bond-length data, see: Allen *et al.* (1987 ▶).
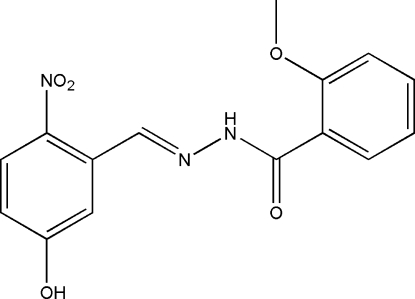


## Experimental

### 

#### Crystal data

C_15_H_13_N_3_O_5_
*M* *_r_* = 315.28
Triclinic, 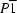

*a* = 8.7540 (9) Å
*b* = 9.0529 (9) Å
*c* = 18.2159 (17) Å
α = 86.902 (5)°
β = 83.023 (5)°
γ = 82.509 (5)°
*V* = 1419.6 (2) Å^3^
*Z* = 4
Mo *K*α radiation
μ = 0.11 mm^−1^
*T* = 298 K
0.17 × 0.15 × 0.15 mm

#### Data collection

Bruker SMART CCD diffractometer
Absorption correction: multi-scan (*SADABS*; Sheldrick, 1996 ▶) *T*_min_ = 0.981, *T*_max_ = 0.983
8779 measured reflections
6066 independent reflections
4236 reflections with *I* > 2σ(*I*)
*R*_int_ = 0.017

#### Refinement

*R*[*F* ^2^ > 2σ(*F* ^2^)] = 0.047
*wR*(*F* ^2^) = 0.123
*S* = 1.04
6066 reflections
425 parameters
2 restraints
H atoms treated by a mixture of independent and constrained refinement
Δρ_max_ = 0.23 e Å^−3^
Δρ_min_ = −0.26 e Å^−3^

### 

Data collection: *SMART* (Bruker, 2002 ▶); cell refinement: *SAINT* (Bruker, 2002 ▶); data reduction: *SAINT*; program(s) used to solve structure: *SHELXS97* (Sheldrick, 2008 ▶); program(s) used to refine structure: *SHELXL97* (Sheldrick, 2008 ▶); molecular graphics: *SHELXTL* (Sheldrick, 2008 ▶); software used to prepare material for publication: *SHELXTL*.

## Supplementary Material

Crystal structure: contains datablocks global, I. DOI: 10.1107/S1600536809043840/wn2358sup1.cif

Structure factors: contains datablocks I. DOI: 10.1107/S1600536809043840/wn2358Isup2.hkl

Additional supplementary materials:  crystallographic information; 3D view; checkCIF report

## Figures and Tables

**Table 1:** Hydrogen-bond geometry (Å, °)

*D*—H⋯*A*	*D*—H	H⋯*A*	*D*⋯*A*	*D*—H⋯*A*
O5—H5⋯O6^i^	0.82	1.91	2.721 (2)	169
O10—H10⋯O1	0.82	1.88	2.689 (2)	168

Symmetry code: (i) 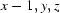.

**Fig. 1.**
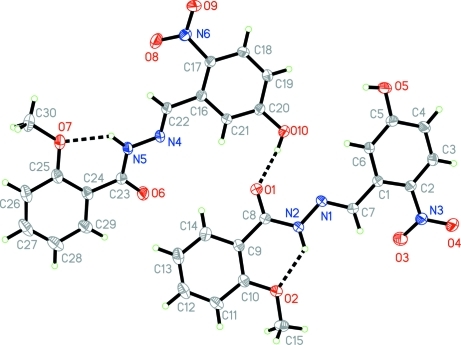
The structure of the asymmetric unit of the title compound, showing the atom-numbering scheme. Displacement ellipsoids are drawn at the 30% probability level. Hydrogen atoms are shown as spheres of arbitrary radius. Hydrogen bonds are shown as dashed lines.

**Fig. 2.**
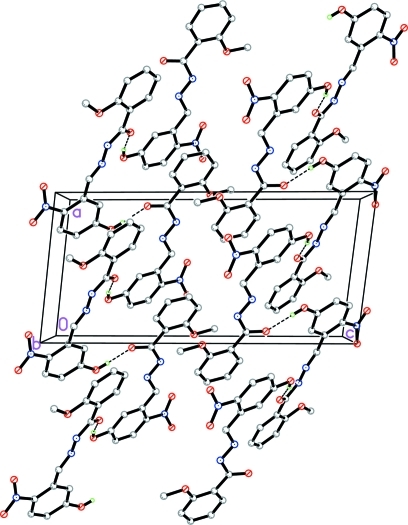
Molecular packing, viewed along the a axis. Hydrogen bonds are shown as dashed lines. H atoms not involved in hydrogen bonding have been omitted.

## supplementary crystallographic information

### Comment

Hydrazone compounds have been of great interest for a long time. These compounds play an important role in biological and structural chemistry (Avaji *et al.*, 2009; Charkoudian *et al.*, 2007; Cukurovali *et al.*, 2006). Recently, we have reported a few hydrazone compounds (Yang, 2008*a*,b,c,d,e, 2007*a*,b,c; Yang & Guo, 2006). As a further investigation in this area, the crystal structure of the new title hydrazone compound is reported.

The asymmetric unit of the title compound, Fig. 1, consists of two independent molecules. Each molecule displays an *E* configuration with respect to the C═N double bond. The dihedral angle between the C1—C6 and C9—C14 benzene rings is 11.1 (2)°, and that between the C16—C21 and C24—C29 benzene rings is 10.9 (2)°. All the bond lengths are within normal ranges (Allen *et al.*, 1987). The C7═N1 and C22═N4 bond lengths of 1.256 (2) and 1.260 (2) Å, respectively, conform to the values for double bonds. The bond length of 1.348 (2) Å between atoms C8 and N2, and that of 1.351 (2) Å between atoms C23 and N5, are intermediate between C—N single bonds and C═N double bonds, because of conjugation effects in the molecules.

In the crystal structure, molecules are linked through intermolecular O—H···O hydrogen bonds (Table 1), forming chains running along the a axis (Fig. 2).

### Experimental

5-Hydroxy-2-nitrobenzaldehyde (0.1 mmol, 16.7 mg) and 2-methoxybenzohydrazide (0.1 mmol, 16.6 mg) were dissolved in MeOH (10 ml). The mixture was stirred at room temperature to give a clear colorless solution. Crystals of the title compound were formed by gradual evaporation of the solvent over a period of 5 days at room temperature.

### Refinement

Nitrogen-bound atoms H2 and H5A were located in a difference Fourier map and refined isotropically, with N—H distances restrained to 0.90 (1) Å. Other H atoms were placed in idealized positions and constrained to ride on their parent atoms, with O—H distances of 0.82 Å, C—H distances of 0.93–0.96 Å, and with *U*_iso_(H) = 1.2*U*_eq_(C) and 1.5*U*_eq_(O and C_methyl_).

### Crystal data

**Table:**

C_15_H_13_N_3_O_5_	*Z* = 4
*M**_r_* = 315.28	*F*(000) = 656
Triclinic, *P*1	*D*_x_ = 1.475 Mg m^−^^3^
Hall symbol: -P 1	Mo *K*α radiation, λ = 0.71073 Å
*a* = 8.7540 (9) Å	Cell parameters from 2340 reflections
*b* = 9.0529 (9) Å	θ = 2.5–25.3°
*c* = 18.2159 (17) Å	µ = 0.11 mm^−^^1^
α = 86.902 (5)°	*T* = 298 K
β = 83.023 (5)°	Block, colorless
γ = 82.509 (5)°	0.17 × 0.15 × 0.15 mm
*V* = 1419.6 (2) Å^3^	

### Data collection

**Table:**

Bruker SMART CCD diffractometer	6066 independent reflections
Radiation source: fine-focus sealed tube	4236 reflections with *I* > 2σ(*I*)
graphite	*R*_int_ = 0.017
ω scans	θ_max_ = 27.0°, θ_min_ = 2.3°
Absorption correction: multi-scan (*SADABS*; Sheldrick, 1996)	*h* = −10→11
*T*_min_ = 0.981, *T*_max_ = 0.983	*k* = −11→9
8779 measured reflections	*l* = −23→23

### Refinement

**Table:**

Refinement on *F*^2^	Primary atom site location: structure-invariant direct methods
Least-squares matrix: full	Secondary atom site location: difference Fourier map
*R*[*F*^2^ > 2σ(*F*^2^)] = 0.047	Hydrogen site location: inferred from neighbouring sites
*wR*(*F*^2^) = 0.123	H atoms treated by a mixture of independent and constrained refinement
*S* = 1.04	*w* = 1/[σ^2^(*F*_o_^2^) + (0.0544*P*)^2^ + 0.1933*P*] where *P* = (*F*_o_^2^ + 2*F*_c_^2^)/3
6066 reflections	(Δ/σ)_max_ = 0.001
425 parameters	Δρ_max_ = 0.23 e Å^−^^3^
2 restraints	Δρ_min_ = −0.26 e Å^−^^3^

### Special details

**Table:**

Geometry. All e.s.d.'s (except the e.s.d. in the dihedral angle between two l.s. planes) are estimated using the full covariance matrix. The cell e.s.d.'s are taken into account individually in the estimation of e.s.d.'s in distances, angles and torsion angles; correlations between e.s.d.'s in cell parameters are only used when they are defined by crystal symmetry. An approximate (isotropic) treatment of cell e.s.d.'s is used for estimating e.s.d.'s involving l.s. planes.
Refinement. Refinement of *F*^2^ against ALL reflections. The weighted *R*-factor *wR* and goodness of fit *S* are based on *F*^2^, conventional *R*-factors *R* are based on *F*, with *F* set to zero for negative *F*^2^. The threshold expression of *F*^2^ > σ(*F*^2^) is used only for calculating *R*-factors(gt) *etc*. and is not relevant to the choice of reflections for refinement. *R*-factors based on *F*^2^ are statistically about twice as large as those based on *F*, and *R*- factors based on ALL data will be even larger.

### Fractional atomic coordinates and isotropic or equivalent isotropic displacement parameters (Å^2^)

**Table:**

	*x*	*y*	*z*	*U*_iso_*/*U*_eq_	
O1	0.43038 (17)	0.56766 (15)	0.20225 (9)	0.0651 (4)	
O2	0.51734 (15)	0.97729 (14)	0.11083 (7)	0.0496 (3)	
O3	0.04575 (17)	0.86066 (17)	−0.04665 (8)	0.0648 (4)	
O4	−0.19271 (18)	0.88756 (16)	−0.06698 (8)	0.0642 (4)	
O5	−0.22760 (16)	0.36644 (15)	0.16573 (7)	0.0516 (4)	
H5	−0.1793	0.3639	0.2017	0.077*	
O6	0.93551 (17)	0.39747 (18)	0.28075 (8)	0.0630 (4)	
O7	1.02232 (16)	0.21547 (16)	0.48449 (7)	0.0548 (4)	
O8	0.54241 (17)	−0.09248 (17)	0.43071 (8)	0.0655 (4)	
O9	0.30249 (18)	−0.12659 (17)	0.44316 (8)	0.0662 (4)	
O10	0.27735 (16)	0.33708 (14)	0.18156 (7)	0.0489 (3)	
H10	0.3249	0.4094	0.1814	0.073*	
N1	0.20958 (17)	0.68586 (16)	0.12448 (8)	0.0415 (4)	
N2	0.32509 (17)	0.76663 (16)	0.13956 (9)	0.0426 (4)	
N3	−0.08536 (19)	0.82546 (17)	−0.03512 (8)	0.0426 (4)	
N4	0.71155 (17)	0.24869 (17)	0.34137 (8)	0.0405 (4)	
N5	0.82807 (18)	0.27753 (18)	0.38088 (8)	0.0437 (4)	
N6	0.41209 (19)	−0.06591 (17)	0.41211 (8)	0.0435 (4)	
C1	−0.02579 (19)	0.67167 (19)	0.07731 (9)	0.0346 (4)	
C2	−0.1160 (2)	0.70609 (19)	0.01894 (9)	0.0357 (4)	
C3	−0.2360 (2)	0.6249 (2)	0.00901 (10)	0.0413 (4)	
H3	−0.2926	0.6485	−0.0310	0.050*	
C4	−0.2708 (2)	0.5109 (2)	0.05763 (10)	0.0433 (4)	
H4	−0.3505	0.4564	0.0507	0.052*	
C5	−0.1862 (2)	0.47659 (19)	0.11782 (9)	0.0378 (4)	
C6	−0.06376 (19)	0.55545 (19)	0.12606 (9)	0.0366 (4)	
H6	−0.0057	0.5296	0.1653	0.044*	
C7	0.1012 (2)	0.7541 (2)	0.09190 (10)	0.0398 (4)	
H7	0.1011	0.8542	0.0773	0.048*	
C8	0.4347 (2)	0.6969 (2)	0.17976 (10)	0.0404 (4)	
C9	0.56063 (19)	0.7798 (2)	0.19878 (10)	0.0380 (4)	
C10	0.6016 (2)	0.9147 (2)	0.16516 (10)	0.0395 (4)	
C11	0.7241 (2)	0.9775 (2)	0.18779 (11)	0.0497 (5)	
H11	0.7510	1.0673	0.1661	0.060*	
C12	0.8050 (2)	0.9064 (3)	0.24229 (12)	0.0584 (6)	
H12	0.8865	0.9492	0.2573	0.070*	
C13	0.7685 (2)	0.7741 (3)	0.27496 (12)	0.0576 (6)	
H13	0.8250	0.7267	0.3115	0.069*	
C14	0.6473 (2)	0.7120 (2)	0.25317 (10)	0.0479 (5)	
H14	0.6224	0.6220	0.2754	0.058*	
C15	0.5651 (2)	1.1071 (2)	0.07190 (12)	0.0562 (5)	
H15A	0.5549	1.1870	0.1054	0.084*	
H15B	0.5011	1.1353	0.0330	0.084*	
H15C	0.6714	1.0866	0.0511	0.084*	
C16	0.47579 (19)	0.15649 (19)	0.33463 (9)	0.0350 (4)	
C17	0.3836 (2)	0.04192 (19)	0.35190 (9)	0.0352 (4)	
C18	0.2637 (2)	0.0237 (2)	0.31102 (10)	0.0404 (4)	
H18	0.2052	−0.0547	0.3231	0.048*	
C19	0.2313 (2)	0.1207 (2)	0.25315 (10)	0.0419 (4)	
H19	0.1525	0.1071	0.2251	0.050*	
C20	0.3170 (2)	0.23981 (19)	0.23648 (9)	0.0373 (4)	
C21	0.43944 (19)	0.25484 (19)	0.27658 (9)	0.0365 (4)	
H21	0.4983	0.3329	0.2641	0.044*	
C22	0.6037 (2)	0.1839 (2)	0.37580 (10)	0.0400 (4)	
H22	0.6048	0.1544	0.4255	0.048*	
C23	0.9387 (2)	0.3552 (2)	0.34549 (10)	0.0413 (4)	
C24	1.0637 (2)	0.3942 (2)	0.38721 (10)	0.0420 (4)	
C25	1.1028 (2)	0.3282 (2)	0.45480 (11)	0.0451 (5)	
C26	1.2213 (2)	0.3780 (3)	0.48783 (13)	0.0611 (6)	
H26	1.2481	0.3351	0.5328	0.073*	
C27	1.2983 (3)	0.4902 (3)	0.45383 (16)	0.0701 (7)	
H27	1.3751	0.5246	0.4770	0.084*	
C28	1.2647 (3)	0.5530 (3)	0.38670 (15)	0.0669 (7)	
H28	1.3199	0.6272	0.3637	0.080*	
C29	1.1482 (2)	0.5044 (2)	0.35392 (12)	0.0531 (5)	
H29	1.1252	0.5464	0.3082	0.064*	
C30	1.0611 (3)	0.1437 (3)	0.55204 (11)	0.0626 (6)	
H30A	1.1698	0.1073	0.5472	0.094*	
H30B	1.0019	0.0617	0.5637	0.094*	
H30C	1.0380	0.2134	0.5909	0.094*	
H2	0.331 (3)	0.8598 (14)	0.1214 (12)	0.080*	
H5A	0.833 (3)	0.243 (2)	0.4276 (7)	0.080*	

### Atomic displacement parameters (Å^2^)

**Table:**

	*U*^11^	*U*^22^	*U*^33^	*U*^12^	*U*^13^	*U*^23^
O1	0.0588 (9)	0.0480 (9)	0.0964 (12)	−0.0217 (7)	−0.0397 (8)	0.0299 (8)
O2	0.0452 (8)	0.0470 (8)	0.0607 (9)	−0.0190 (6)	−0.0160 (6)	0.0159 (6)
O3	0.0507 (9)	0.0761 (11)	0.0666 (10)	−0.0205 (8)	−0.0020 (7)	0.0268 (8)
O4	0.0730 (10)	0.0556 (9)	0.0715 (10)	−0.0181 (7)	−0.0394 (8)	0.0242 (7)
O5	0.0604 (9)	0.0534 (8)	0.0477 (8)	−0.0284 (7)	−0.0168 (7)	0.0145 (6)
O6	0.0574 (9)	0.0893 (11)	0.0492 (9)	−0.0331 (8)	−0.0201 (7)	0.0254 (8)
O7	0.0522 (8)	0.0695 (10)	0.0476 (8)	−0.0127 (7)	−0.0238 (7)	0.0083 (7)
O8	0.0525 (9)	0.0743 (10)	0.0677 (10)	−0.0008 (7)	−0.0205 (8)	0.0273 (8)
O9	0.0741 (11)	0.0728 (10)	0.0595 (9)	−0.0395 (8)	−0.0200 (8)	0.0271 (8)
O10	0.0550 (9)	0.0468 (8)	0.0502 (8)	−0.0150 (6)	−0.0250 (6)	0.0149 (6)
N1	0.0358 (8)	0.0410 (9)	0.0513 (9)	−0.0146 (7)	−0.0137 (7)	0.0082 (7)
N2	0.0386 (8)	0.0378 (8)	0.0558 (10)	−0.0144 (7)	−0.0182 (7)	0.0112 (7)
N3	0.0461 (9)	0.0428 (9)	0.0404 (9)	−0.0097 (7)	−0.0102 (7)	0.0051 (7)
N4	0.0372 (8)	0.0488 (9)	0.0391 (8)	−0.0115 (7)	−0.0156 (7)	0.0050 (7)
N5	0.0394 (9)	0.0598 (10)	0.0360 (8)	−0.0171 (7)	−0.0143 (7)	0.0085 (7)
N6	0.0506 (10)	0.0404 (9)	0.0406 (9)	−0.0082 (7)	−0.0098 (7)	0.0053 (7)
C1	0.0298 (9)	0.0360 (9)	0.0385 (9)	−0.0057 (7)	−0.0052 (7)	0.0017 (7)
C2	0.0378 (9)	0.0355 (9)	0.0339 (9)	−0.0055 (7)	−0.0052 (7)	0.0022 (7)
C3	0.0422 (10)	0.0476 (11)	0.0368 (10)	−0.0089 (8)	−0.0142 (8)	0.0024 (8)
C4	0.0435 (11)	0.0459 (11)	0.0450 (11)	−0.0171 (8)	−0.0119 (8)	0.0011 (8)
C5	0.0410 (10)	0.0360 (9)	0.0365 (9)	−0.0083 (8)	−0.0029 (8)	0.0022 (7)
C6	0.0337 (9)	0.0405 (10)	0.0371 (9)	−0.0076 (7)	−0.0097 (7)	0.0043 (7)
C7	0.0388 (10)	0.0363 (10)	0.0458 (10)	−0.0105 (8)	−0.0100 (8)	0.0091 (8)
C8	0.0368 (10)	0.0391 (10)	0.0471 (11)	−0.0101 (8)	−0.0108 (8)	0.0078 (8)
C9	0.0314 (9)	0.0426 (10)	0.0409 (10)	−0.0061 (7)	−0.0060 (7)	−0.0028 (8)
C10	0.0325 (9)	0.0446 (10)	0.0422 (10)	−0.0068 (8)	−0.0044 (8)	−0.0033 (8)
C11	0.0421 (11)	0.0505 (12)	0.0602 (13)	−0.0177 (9)	−0.0063 (9)	−0.0070 (10)
C12	0.0470 (12)	0.0711 (15)	0.0638 (14)	−0.0138 (11)	−0.0193 (10)	−0.0179 (12)
C13	0.0543 (13)	0.0689 (15)	0.0540 (13)	−0.0043 (11)	−0.0241 (10)	−0.0102 (11)
C14	0.0476 (11)	0.0512 (12)	0.0459 (11)	−0.0042 (9)	−0.0124 (9)	0.0013 (9)
C15	0.0533 (13)	0.0510 (12)	0.0652 (14)	−0.0200 (10)	−0.0035 (10)	0.0141 (10)
C16	0.0319 (9)	0.0398 (10)	0.0327 (9)	−0.0039 (7)	−0.0040 (7)	0.0022 (7)
C17	0.0369 (9)	0.0353 (9)	0.0331 (9)	−0.0036 (7)	−0.0058 (7)	0.0025 (7)
C18	0.0431 (10)	0.0391 (10)	0.0417 (10)	−0.0153 (8)	−0.0075 (8)	0.0032 (8)
C19	0.0421 (10)	0.0451 (11)	0.0421 (10)	−0.0109 (8)	−0.0149 (8)	0.0007 (8)
C20	0.0386 (10)	0.0392 (10)	0.0341 (9)	−0.0034 (8)	−0.0080 (7)	0.0040 (7)
C21	0.0350 (9)	0.0379 (10)	0.0383 (9)	−0.0094 (7)	−0.0081 (7)	0.0050 (7)
C22	0.0385 (10)	0.0479 (11)	0.0351 (9)	−0.0090 (8)	−0.0116 (8)	0.0084 (8)
C23	0.0371 (10)	0.0455 (11)	0.0426 (10)	−0.0073 (8)	−0.0096 (8)	0.0045 (8)
C24	0.0312 (9)	0.0459 (11)	0.0498 (11)	−0.0020 (8)	−0.0087 (8)	−0.0084 (9)
C25	0.0348 (10)	0.0507 (11)	0.0509 (12)	−0.0012 (9)	−0.0104 (8)	−0.0107 (9)
C26	0.0478 (12)	0.0715 (15)	0.0688 (15)	−0.0012 (11)	−0.0264 (11)	−0.0182 (12)
C27	0.0467 (13)	0.0724 (16)	0.099 (2)	−0.0132 (12)	−0.0231 (13)	−0.0300 (15)
C28	0.0499 (13)	0.0615 (15)	0.0938 (19)	−0.0185 (11)	−0.0072 (13)	−0.0183 (13)
C29	0.0434 (11)	0.0504 (12)	0.0667 (14)	−0.0097 (9)	−0.0041 (10)	−0.0077 (10)
C30	0.0601 (14)	0.0785 (16)	0.0502 (12)	0.0022 (12)	−0.0255 (10)	0.0047 (11)

### Geometric parameters (Å, °)

**Table:**

O1—C8	1.223 (2)	C9—C10	1.405 (3)
O2—C10	1.361 (2)	C10—C11	1.391 (2)
O2—C15	1.423 (2)	C11—C12	1.373 (3)
O3—N3	1.2217 (19)	C11—H11	0.9300
O4—N3	1.2228 (19)	C12—C13	1.367 (3)
O5—C5	1.346 (2)	C12—H12	0.9300
O5—H5	0.8200	C13—C14	1.371 (3)
O6—C23	1.223 (2)	C13—H13	0.9300
O7—C25	1.366 (2)	C14—H14	0.9300
O7—C30	1.418 (2)	C15—H15A	0.9600
O8—N6	1.220 (2)	C15—H15B	0.9600
O9—N6	1.231 (2)	C15—H15C	0.9600
O10—C20	1.344 (2)	C16—C21	1.385 (2)
O10—H10	0.8200	C16—C17	1.395 (2)
N1—C7	1.256 (2)	C16—C22	1.475 (2)
N1—N2	1.3837 (19)	C17—C18	1.390 (2)
N2—C8	1.348 (2)	C18—C19	1.367 (2)
N2—H2	0.895 (10)	C18—H18	0.9300
N3—C2	1.451 (2)	C19—C20	1.393 (2)
N4—C22	1.260 (2)	C19—H19	0.9300
N4—N5	1.3764 (19)	C20—C21	1.393 (2)
N5—C23	1.351 (2)	C21—H21	0.9300
N5—H5A	0.895 (10)	C22—H22	0.9300
N6—C17	1.451 (2)	C23—C24	1.493 (2)
C1—C6	1.387 (2)	C24—C29	1.388 (3)
C1—C2	1.399 (2)	C24—C25	1.400 (3)
C1—C7	1.474 (2)	C25—C26	1.394 (3)
C2—C3	1.392 (2)	C26—C27	1.371 (3)
C3—C4	1.364 (2)	C26—H26	0.9300
C3—H3	0.9300	C27—C28	1.368 (3)
C4—C5	1.396 (2)	C27—H27	0.9300
C4—H4	0.9300	C28—C29	1.373 (3)
C5—C6	1.390 (2)	C28—H28	0.9300
C6—H6	0.9300	C29—H29	0.9300
C7—H7	0.9300	C30—H30A	0.9600
C8—C9	1.495 (2)	C30—H30B	0.9600
C9—C14	1.389 (3)	C30—H30C	0.9600
			
C10—O2—C15	117.91 (15)	C13—C14—C9	121.9 (2)
C5—O5—H5	109.5	C13—C14—H14	119.1
C25—O7—C30	118.82 (15)	C9—C14—H14	119.1
C20—O10—H10	109.5	O2—C15—H15A	109.5
C7—N1—N2	117.33 (15)	O2—C15—H15B	109.5
C8—N2—N1	117.45 (14)	H15A—C15—H15B	109.5
C8—N2—H2	120.6 (15)	O2—C15—H15C	109.5
N1—N2—H2	121.8 (15)	H15A—C15—H15C	109.5
O3—N3—O4	122.64 (16)	H15B—C15—H15C	109.5
O3—N3—C2	118.98 (15)	C21—C16—C17	117.22 (15)
O4—N3—C2	118.38 (15)	C21—C16—C22	118.02 (16)
C22—N4—N5	117.22 (15)	C17—C16—C22	124.70 (15)
C23—N5—N4	117.46 (15)	C18—C17—C16	121.56 (15)
C23—N5—H5A	121.0 (15)	C18—C17—N6	117.20 (15)
N4—N5—H5A	121.5 (16)	C16—C17—N6	121.22 (15)
O8—N6—O9	122.25 (16)	C19—C18—C17	120.27 (16)
O8—N6—C17	119.37 (16)	C19—C18—H18	119.9
O9—N6—C17	118.38 (15)	C17—C18—H18	119.9
C6—C1—C2	117.02 (15)	C18—C19—C20	119.51 (16)
C6—C1—C7	118.44 (15)	C18—C19—H19	120.2
C2—C1—C7	124.49 (15)	C20—C19—H19	120.2
C3—C2—C1	121.54 (16)	O10—C20—C21	122.29 (16)
C3—C2—N3	117.21 (15)	O10—C20—C19	118.01 (15)
C1—C2—N3	121.23 (15)	C21—C20—C19	119.70 (16)
C4—C3—C2	120.29 (16)	C16—C21—C20	121.65 (16)
C4—C3—H3	119.9	C16—C21—H21	119.2
C2—C3—H3	119.9	C20—C21—H21	119.2
C3—C4—C5	119.66 (16)	N4—C22—C16	117.72 (16)
C3—C4—H4	120.2	N4—C22—H22	121.1
C5—C4—H4	120.2	C16—C22—H22	121.1
O5—C5—C6	122.68 (16)	O6—C23—N5	121.09 (16)
O5—C5—C4	117.72 (16)	O6—C23—C24	119.70 (17)
C6—C5—C4	119.59 (16)	N5—C23—C24	119.17 (16)
C1—C6—C5	121.84 (16)	C29—C24—C25	118.61 (17)
C1—C6—H6	119.1	C29—C24—C23	115.53 (17)
C5—C6—H6	119.1	C25—C24—C23	125.85 (18)
N1—C7—C1	118.35 (16)	O7—C25—C26	123.40 (19)
N1—C7—H7	120.8	O7—C25—C24	117.22 (16)
C1—C7—H7	120.8	C26—C25—C24	119.4 (2)
O1—C8—N2	120.76 (16)	C27—C26—C25	119.8 (2)
O1—C8—C9	119.55 (16)	C27—C26—H26	120.1
N2—C8—C9	119.67 (15)	C25—C26—H26	120.1
C14—C9—C10	118.09 (16)	C28—C27—C26	121.6 (2)
C14—C9—C8	115.65 (16)	C28—C27—H27	119.2
C10—C9—C8	126.22 (16)	C26—C27—H27	119.2
O2—C10—C11	123.15 (17)	C27—C28—C29	118.8 (2)
O2—C10—C9	117.06 (15)	C27—C28—H28	120.6
C11—C10—C9	119.79 (17)	C29—C28—H28	120.6
C12—C11—C10	119.68 (19)	C28—C29—C24	121.7 (2)
C12—C11—H11	120.2	C28—C29—H29	119.1
C10—C11—H11	120.2	C24—C29—H29	119.1
C13—C12—C11	121.47 (19)	O7—C30—H30A	109.5
C13—C12—H12	119.3	O7—C30—H30B	109.5
C11—C12—H12	119.3	H30A—C30—H30B	109.5
C12—C13—C14	119.1 (2)	O7—C30—H30C	109.5
C12—C13—H13	120.5	H30A—C30—H30C	109.5
C14—C13—H13	120.5	H30B—C30—H30C	109.5

### Hydrogen-bond geometry (Å, °)

**Table:**

*D*—H···*A*	*D*—H	H···*A*	*D*···*A*	*D*—H···*A*
O5—H5···O6^i^	0.82	1.91	2.721 (2)	169
O10—H10···O1	0.82	1.88	2.689 (2)	168

Symmetry codes: (i) *x*−1, *y*, *z*.
